# Addition/Correction to ”Advancing Sewage Sludge Valorization: Sustainable Biofuel Production through First-Principles Modeling and Process Simulation”

**DOI:** 10.1021/acs.iecr.5c00861

**Published:** 2025-03-27

**Authors:** Francesco Negri, Francesco Gallo, Flavio Manenti

**Affiliations:** † Itelyum Regeneration S.p.A., Via Tavernelle 19, Pieve Fissiraga 26854, Italy; ‡ Dipartimento di Chimica, Materiali e Ingegneria Chimica ”Giulio Natta”, Politecnico di Milano, Piazza Leonardo da Vinci 32, Milano 20133, Italy

The authors have provided a new set of Supporting Information, to maximize compatibility among users. Supporting Information now includes Aspen HYSYS simulation files in different formats, with improved convergence behavior. Furthermore, a revised TOC Graphic created according to the official ACS Guidelines has been provided. The revised TOC Graphic is entirely original, composed of unpublished artwork created by the authors.^1^

**Figure 1:**
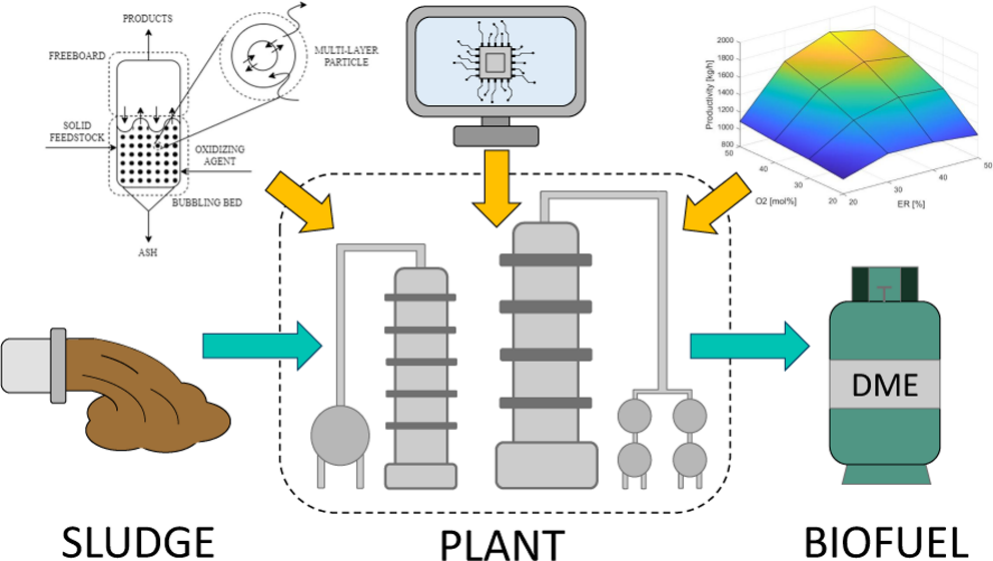


## Supporting Information Available

Process simulation for bio-DME production developed in Aspen HYSYS software, available in multiple file formats (ZIP)
