# Supplementary material for: Addition/Correction to ”Advancing Sewage Sludge Valorization: Sustainable Biofuel Production through First-Principles Modeling and Process Simulation”
Source: Ind Eng Chem Res. 2025 Mar 27;64(14):7617. doi: 10.1021/acs.iecr.5c00861 (PMC11987013; doi:10.1021/acs.iecr.5c00861)
Supplement: Supplementary file 2 — ie5c00861_si_002.pdf [file ie5c00861_si_002.pdf]

Supporting Information

Advancing Sewage Sludge Valorization: Sustainable Biofuel Production through First-Principles Modeling and Process Simulation

Francesco Negri,[†,‡] Francesco Gallo,[†] and Flavio Manenti[*,‡]

†*Itelyum Regeneration S.p.A., Via Tavernelle, 19, Pieve Fissiraga, 26854, Italy*

‡*Dipartimento di Chimica, Materiali e Ingegneria Chimica "Giulio Natta", Politecnico di Milano, Piazza Leonardo da Vinci 32, Milano, 20133, Italy*

E-mail: flavio.manenti@polimi.it

Supporting information for the paper is available. It includes a process simulation file for bio-DME production developed in Aspen HYSYS software, available in different formats to maximize compatibility with users (.hsc, .bk0, .xml extensions are available).
